# Clinical characteristics and prognosis of Glioblastoma patients with infratentorial recurrence

**DOI:** 10.1186/s12883-022-03047-9

**Published:** 2023-01-07

**Authors:** Daisuke Kawauchi, Makoto Ohno, Mai Honda-Kitahara, Yasuji Miyakita, Masamichi Takahashi, Shunsuke Yanagisawa, Yukie Tamura, Miyu Kikuchi, Koichi Ichimura, Yoshitaka Narita

**Affiliations:** 1 Department of Neurosurgery and Neuro-Oncology, National Cancer Center Hospital, 5-1-1, Tsukiji, Chuo-Ku, Tokyo, 104-0045 Japan; 2 Department of Brain Disease Translational Research, Faculty of Medicine, Juntendo University, Tokyo, Japan

**Keywords:** Infratentorial, Glioblastoma, Recurrence

## Abstract

**Background:**

Glioblastoma (GBM) infrequently recurs in the infratentorial region. Such Infratentorial recurrence (ITR) has some clinically unique characteristics, such as presenting unspecific symptoms and providing patients a chance to receive additional radiotherapy. However, the clinical significances of ITR are not well studied.

**Methods:**

We reviewed newly diagnosed isocitrate dehydrogenase (IDH)-wildtype GBM patients treated at our institution between October 2008 and December 2018. ITR was defined as any type of recurrence in GBM, including dissemination or distant recurrence, which primarily developed in the supratentorial region and recurred in the infratentorial region.

**Results:**

Of 134 patients with newly diagnosed IDH-wildtype GBM, six (4.5%) were classified as having ITR. There was no significant difference in median duration from the first surgery to ITR development between patients with and without ITR (12.2 vs. 10.2 months, *P* = 0.65). The primary symptoms of ITR were gait disturbance (100%, *n* = 6), dizziness (50.0%, *n* = 3), nausea (33.3%, *n* = 2), and cerebellar mutism (16.7%, *n* = 1). In four cases (66.7%), symptoms were presented before ITR development. All patients received additional treatments for ITR. The median post-recurrence survival (PRS) of ITR patients was significantly shorter than that of general GBM patients (5.5 vs. 9.1 months, *P* = 0.023). However, chemoradiotherapy contributed to palliating symptoms such as nausea.

**Conclusions:**

ITR is a severe recurrence type in GBM patients. Its symptoms are neurologically unspecific and can be overlooked or misdiagnosed as side effects of treatments. Carefully checking the infratentorial region, especially around the fourth ventricle, is essential during the GBM patient follow-up.

## Background

Glioblastoma (GBM) is the most life-threatening malignant brain tumor and is categorized as a grade 4 tumor by the World Health Organization (WHO). Even with the best treatment of maximal safe surgical resection following chemoradiotherapy with temozolomide (TMZ), recurrence is inevitable in most cases. The majority of GBM recurs locally (75–80%) [1, 2], and such local recurrence is related to shorter survival [2, 3]. GBM infrequently relapses in the anatomically distant region, such as the contralateral hemisphere (4%) [4]. Supratentorial GBM also rarely recurs in the infratentorial region. Infratentorial recurrence (ITR) often demonstrates neurologically non-specific symptoms, including intractable vomiting [5], dizziness, and gait disturbance. Therefore, symptoms of ITR are often overlooked or misdiagnosed as side effects of GBM treatments.

In contrast, GBM patients with ITR have the opportunity to receive additional radiotherapy because the infratentorial region is often outside of the irradiation field of the primary lesion. Despite these unique characteristics, to our knowledge, ITR cases in GBM patients have not been studied in detail. The incidence, symptoms, treatment response, and prognosis of patients with ITR are poorly understood. We gathered ITR cases treated at our institution over the past decade and investigated their clinical significance.

## Methods

### Patient characteristics

This study was a single-center retrospective analysis of a consecutive series of patients with isocitrate dehydrogenase (IDH)-wildtype GBM. First, we identified adult patients with supratentorial GBM (20 to 80 years old) who were newly diagnosed and treated at our institution between October 2008 and December 2018. Patients with H3 histone, family 3A (*H3F3A*), or serine/threonine kinase B-RAF (*BRAF*) mutations were excluded. The patients we reviewed had at least six months of postoperative follow-up, with magnetic resonance imaging performed at least every two months. We collected patient data, including age, sex, clinical history, presurgical physical assessment, radiological images, surgical reports, and postsurgical clinical courses. Histological diagnosis of primary GBM tumor was certified based on the World Health Organization (WHO) classification 2007/2016 of tumors of the central nervous system. In this study, we conformed to WHO classification 2021 and included only IDH-wildtype GBM [6]. Second, we specified GBM patients who developed an ITR. The ITR was defined as any type of GBM recurrence, including dissemination or distant recurrence, in the infratentorial region as the first recurrent site. The ITR was radiologically diagnosed as a gadolinium-enhanced lesion on T1-weighted images or high signal intensity lesions on fluid-attenuated inversion recovery (FLAIR) images.

Molecular profiles of the tumors, including IDH mutations, telomerase reverse transcriptase (*TERT*) promoter mutations, and O6-methylguanine-DNA-methyltransferase (*MGMT*) promoter methylation status, were extracted from medical records. We determined the extent of resection based on the surgeon’s operative notes and on postoperative imaging, classified as either total if 100% of the enhanced lesion was resected, subtotal if 95–99% was resected, partial if < 94% was resected, or a biopsy.

### Molecular analysis

DNA samples were extracted from fresh frozen tumor tissues for all cases using a DNeasy Blood & Tissue Kit (Qiagen, Tokyo, Japan). The presence of hotspot mutations in *IDH1* (R132) and *IDH2* (R172) was assessed by pyrosequencing as previously described [7]. Pyrosequencing assays were designed to detect all known mutations in these codons [7]. As previously reported, the two mutation hotspots in the *TERT* promoter were analyzed in all tumors using Sanger sequencing and/or pyrosequencing [8]. The methylation status of the *MGMT* promoter was analyzed using bisulfite modification of tumor genomic DNA, followed by pyrosequencing, as previously described [8]. Methylation was considered positive when its mean level at the 16 CpG sites was > 16% [8, 9].

### Statistical analysis

Overall survival (OS) was defined as the interval between the initial surgery and death. Progression-free survival (PFS) was defined as the interval between the initial surgery date and the detection of any progression. Thus, in ITR cases, PFS is equivalent to the duration from the initial surgery to ITR development. Post-recurrence survival (PRS) was defined as the interval between the first recurrence and death or last follow-up. Therefore, PRS is equivalent to the difference between PFS and OS. Patients with unknown survival were censored at the last follow-up date. Patient survival was calculated using the Kaplan–Meier method and compared using the log-rank test. Statistical analyses were performed using GraphPad Prism 9 (GraphPad Software, Inc., La Jolla, CA, USA). Statistical significance was defined as *P* < 0.05.

### Ethics approval

This retrospective study used data obtained for clinical purposes. This study was approved by the internal review board of the National Cancer Center (approval number: 2004–066).

## Results

### Patient demographics and initial tumor characteristics

We identified 134 newly diagnosed IDH-wild-type GBM patients at our institution between October 2008 and December 2018. Six (4.5%) patients developed ITR. Table 1 is a list of the six patients with ITR. Four were men, and two were women with a median age of 67 (44–74 years).

**Table 1:** Characteristics of primary tumors of patients with infratentorial recurrence

Patient	Gender	Age	Primary tumor	EOR	Intraoperative ventricle opening	Radiotherapy	Chemotherapy	IDH	TERT promoter	MGMT methylation
1	Female	44	Left frontal lobe	Subtotal	Yes	LBRT 60 Gy/30Fr	TMZ	WT	WT	0%, Low
2	Female	65	Left frontal lobe	Partial	Yes	LBRT 60 Gy/30Fr	TMZ	WT	WT	32.6%, High
3	Male	74	Left parietal lobe	Total	Yes	LBRT 60 Gy/30Fr	TMZ	WT	WT	1.3%, Low
4	Male	62	Left thalamus	Biopsy	No	LBRT 60 Gy/30Fr	TMZ	WT	WT	3.7%, Low
5	Male	69	Right temporal lobe	Subtotal	Yes	LBRT 60 Gy/30Fr	TMZ Nivolumab	WT	C228T	39.9%, High
6	Male	73	Left temporal lobe	Total	Yes	LBRT 60 Gy/30Fr	TMZ	WT	C228T	0.8%, Low

*EOR* Extent of resection, *IDH* Isocitrate dehydrogenase, *LBRT* Local brain radiotherapy, *MGMT* O6-methylguanine-DNA-methyltransferase, *TERT* Telomerase reverse transcriptase, *TMZ* Temozolomide

**Fig. 1:**
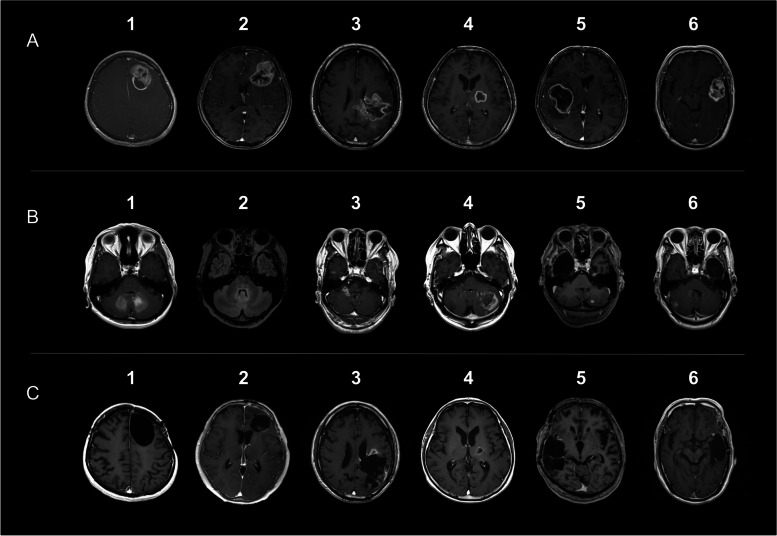
Axial gadolinium-enhanced T1-weighted images of patients with infratentorial recurrence (ITR). **A** Representative images of the initial tumors. **B** Representative images of the ITR. Image of the patient 2 is fluid-attenuated inversion recovery images. **C** Images of the cavities of the initial tumors at ITR development

Representative images of primary tumors are summarized in Fig. 1A. All the initial GBMs exhibited ring-enhanced lesions. Five tumors (83.3%) were on the left, and one was on the right. They were located in the temporal lobe (33.3%, *n* = 2), frontal lobe (33.3%, *n* = 2), parietal lobe (16.7%, *n* = 1), and thalamus (16.7%, *n* = 1). The mean volume of six preoperative enhanced tumor lesions was 28.4 cm^3^ (9.2–42.7 cm^3^).

Five patients (83.3%) underwent tumor resection by craniotomy at the initial presentation under general anesthesia, and one underwent a biopsy for a thalamic lesion. The extent of resection was total in two cases (33.3%), subtotal in two patients (33.3%), partial in one case (16.7%), and biopsy in one case (16.7%). Intraoperative ventricle opening was observed along with tumor resection in five patients (83.3%).

The molecular genetic examination was performed in all cases of initial tumors. Two tumors (33.3%) had *TERT* promoter mutation, and four (66.7%) had low *MGMT* promoter methylation status (cut-off value: 16.0%). All patients received adjuvant local brain radiation of 60 Gy in 30 fractions and chemotherapy with TMZ. Patient 5 received nivolumab in addition to TMZ.

### Characteristics and outcomes of ITRs

Characteristics and prognosis of ITR are listed in Table 2. The median duration from the initial GBM surgery to ITR diagnosis (PFS) was 12.2 months (8.2–16.1 months). The most observed symptom at ITR development was gait disturbance (100.0%, *n* = 6), followed by dizziness (50.0%, *n* = 3), nausea (33.3%, *n* = 2), and cerebellar mutism (16.7%, *n* = 1). The median KPS score at ITR development was 60 (40–70).

**Table 2:** Characteristics and prognosis of patients with infratentorial recurrence (ITR)

Patient	ITR location	Symptoms at ITR diagnosis	KPS	Surgery and Radiotherapy	Chemotherapy	PFS (M)	OS (M)	PRS (M)
1	Fourth ventricle	Gait disturbance, dizziness, nausea, cerebellar mutism	70	LBRT 60 Gy/30Fr	TMZ	14.3	21.9	7.6
2	Fourth ventricle	Gait disturbance, dizziness, nausea	40	LBRT 25 Gy/5Fr	Bev	11.2	14.0	2.7
3	Right Flocculus Fourth ventricle	Gait disturbance	50	None	TMZ + Procarbazine	8.2	11.7	3.4
4	Left cerebellar hemisphere	Gait disturbance	70	Surgery + LBRT 60 Gy/30Fr	TMZ	12.1	19.7	7.6
5	Left cerebellar hemisphere	Gait disturbance, dizziness	70	LBRT 25 Gy/5Fr	TMZ + Bev	16.1	24.7	8.6
6	Fourth ventricle Right cerebellar hemisphere Left tectum of midbrain	Gait disturbance	50	Cerebellum to whole spine 36 Gy/20Fr	Bev	12.2	15.1	2.9

*Bev* Bevacizumab, *KPS* Karnofsky performance status, *LBRT* Local brain radiotherapy, *OS* Overall survival, *PFS* Progression free survival, *PRS* Post-recurrence survival, *TMZ* Temozolomide

The radiological images of the ITR and postoperative tumor cavity at ITR development are summarized in Figs. 1B and C. ITRs were found in the fourth ventricle in four cases (66.7%), in the cerebellum hemisphere in three patients (50.0%), in flocculus in one case (16.7%) and the tectum of the midbrain in one case (16.7%).

The treatments for ITR included surgery following chemoradiotherapy (16.7%, *n* = 1), chemoradiotherapy (66.7%, *n* = 4), and chemotherapy alone (16.7%, *n* = 1). TMZ (66.7%, *n* = 4) and bevacizumab (50.0%, *n* = 3) were the most commonly used therapeutic agents for ITR. The most applied radiation dose was 25 Gy in 5 fractions (33.3%, *n* = 2) or 60 Gy in 30 fractions (33.3%, *n* = 2). No severe adverse effects due to chemoradiotherapy were documented. Two (33.3%) patients with fourth ventricular ITR (patients 1 and 2) presented nausea and intractable vomiting upon ITR treatment. While serotonin 5-HT_3_ receptor antagonists had limited efficacy in relieving the symptom, chemoradiotherapy relieved the intractable vomiting within one or two weeks. Gait disturbance was also observed in all patients and did not improve with any treatment. Patient 1 suffered from cerebellar mutism. These symptoms largely contributed to worse patient performance status.

**Fig. 2:**
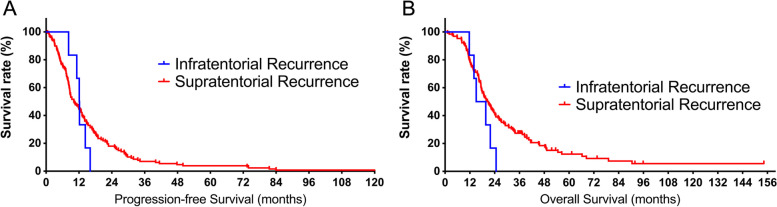
Kaplan–Meier curves of progression-free survival (PFS) and overall survival (OS). **A** The median PFS of patients with and without infratentorial recurrence (ITR) was 12.2 vs. 10.2 months, respectively (*P* = 0.65). **B** The median OS of patients with and without ITR was 17.4 vs. 20.8 months, respectively (*P* = 0.13)

**Fig. 3:**
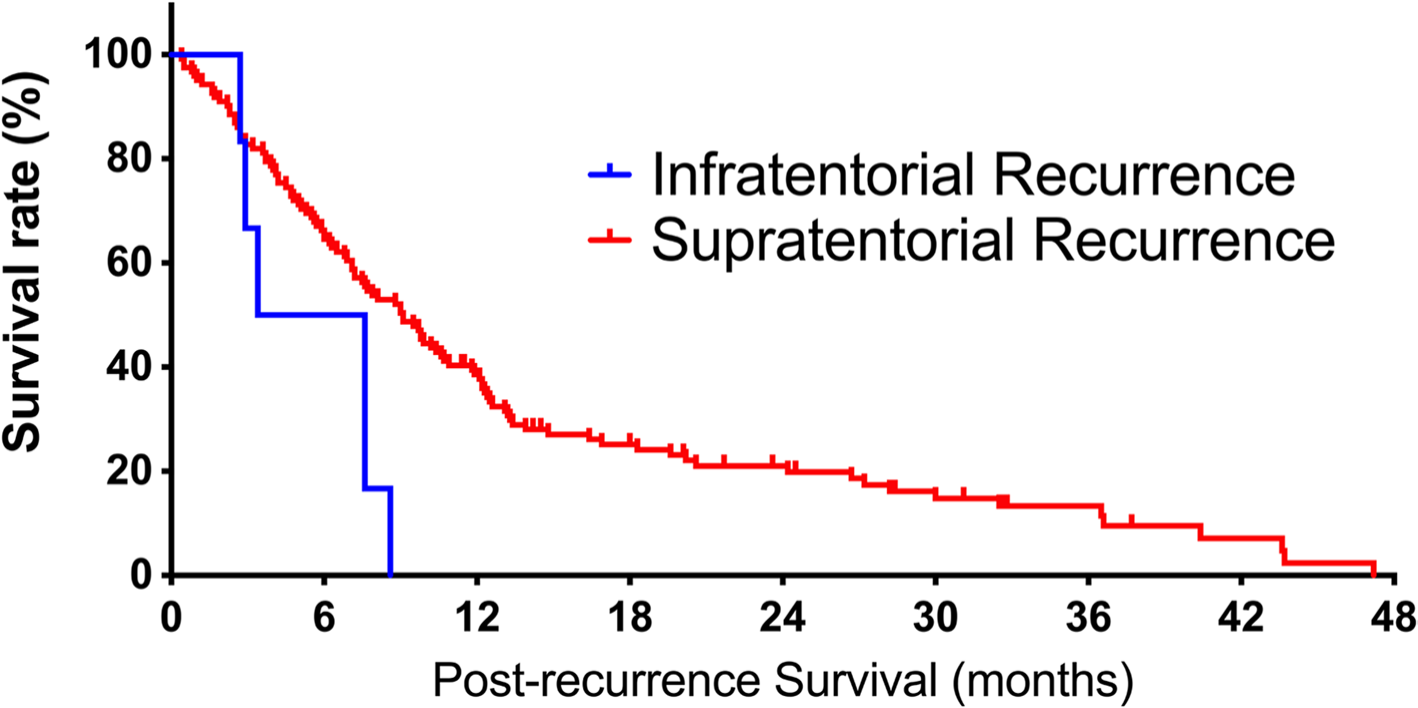
Kaplan–Meier curves of post-recurrence survival (PRS). The median PRS of patients with and without infratentorial recurrence (ITR) was 5.5 vs 9.1 months, respectively (*P* = 0.023)

There was no significant difference in PFS (12.2 vs. 10.2 months, respectively, *P* = 0.65, Fig. 2A) and OS (17.4 vs. 20.8 months, respectively, *P* = 0.13, Fig. 2B) between GBM patients with and without ITR. In contrast, the PRS of GBM patients with ITR demonstrated significantly shorter survival than those without ITR (5.5 vs. 9.1 months, respectively, *P* = 0.023, Fig. 3).

## Discussion

As expected, radiologically diagnosed ITR was a rare complication (4.5%) in GBM patients. Also, this incidence of ITR was close to that of distant recurrence in the contralateral hemisphere (4%) previously reported. [4]. In contrast, a recent autopsy study revealed that extensive GBM infiltration of the brainstem was observed in 67% of patients [10]. This result indicates that ITR may be a more frequent complication at a microscopic level.

The pathogenesis of ITR in patients with GBM remains unclear. The presumed mechanism includes distant recurrence through fiber tracts and leptomeningeal spread via the cerebrospinal fluid. For example, ITR locates in the dentate nucleus in patient 4. The dentate nucleus is the origin of the dentatorubrothalamic tract, which terminates in the contralateral thalamus [11]. Also, thalamocortical radiations connect the thalamus to various areas of the cerebral cortex [12]. Since malignant glioma cells spread and migrate along white matter [13], tumor cells might have migrated from supratentorial to infratentorial regions along white matter tracts through dentate nuclei.

In contrast, in patients 2, 3, and 6, tumors relapsed on a fourth ventricular wall or basal cistern. These recurrent patterns imply leptomeningeal dissemination (LMD) as the pathogenesis of ITR. Previous studies have reported that supratentorial GBM cells metastasize into the fourth ventricle or cerebellum via the cerebrospinal fluid (CSF) through an aqueduct from the third or lateral ventricle [5, 14]. Moreover, tumor cells may disseminate through the CSF with a predilection to regions with slow CSF flow or gravity-dependent sites, such as the basal cisterns or posterior fossa [15]. In clinical situations, however, the diagnosis of LMD based on CSF cytology is challenging due to its low sensitivity (25–45%) [16, 17].

One advantage of ITR is that patients have a chance to receive additional radiotherapy. Unlike local recurrence, the infratentorial region is often outside the primary lesion's irradiation field. In this study, five out of six patients received additional radiotherapy. This study administered three doses (25, 36, and 60 Gy). Unfortunately, the efficacy of radiochemotherapy against ITR was very limited. The median PRS of ITR patients was 5.5 months and was significantly shorter than that of general GBM patients. This poor prognosis is comparable to that of LMD patients (2.1–5.7 months) [16–20]. This result demonstrates that ITR is a severe pattern of GBM recurrence compared to local recurrence.

More than half (57%) of GBM patients presented focal symptoms as an initial indication of GBM [21]. In contrast, the symptoms observed in ITR were mostly neurologically unspecific, such as gait disturbance and nausea. These unspecific symptoms can easily be misdiagnosed as side effects of chemotherapy and can be observed on the left side. Four cases (66.7%) exhibited new symptoms several weeks before the MRI examination. Thus, ITR must be considered when patients present with neurologically non-focused symptoms, especially gait disturbance.

Moreover, patients with ITR often experience persistent nausea and intractable vomiting, leading to appetite loss. Cohen et al. have reported three cases of uncontrollable vomiting from a GBM that disseminated to the fourth ventricle [5]. In these cases, additional irradiation to the infratentorial region achieved complete remission of symptoms. Here, patients 1 and 2, who developed ITR in the fourth ventricle, experienced persistent nausea. Although 5-HT_3_ receptor antagonists have minimal efficacy, chemoradiation therapy helped to relieve the patients’ intractable nausea. The prompt introduction of chemoradiotherapy is key to maintaining the quality of life in ITR patients.

The main limitation of this study was the small sample size due to the rarity of ITR; therefore, our results need to be carefully interpreted. Another significant limitation is that the therapeutic strategies employed may be biased based on patient performance status. Since this was a retrospective study, patients with a good performance status might have received more intensive treatment, and those with a poor performance status might have undergone more palliative treatment. These therapeutic differences reflect realistic clinical decisions, although they hinder the objective assessment of outcomes in patients with ITR.

## Conclusions

ITR is a severe type of recurrence in GBM patients. Its symptoms are neurologically unspecific and can be overlooked or misdiagnosed as side effects of treatments. Carefully checking the infratentorial region, especially around the fourth ventricle, is essential during the GBM patient follow-up.

## Data Availability

The datasets used and/or analyzed during the current study are available from the corresponding author on reasonable request.

## Abbreviations

BRAF: Serine/threonine kinase B-RAF
CSF: Cerebrospinal fluid
FLAIR: Fluid-attenuated inversion recovery
GBM: Glioblastoma
H3F3A: H3 histone, family 3A
IDH: Isocitrate dehydrogenase
ITR: Infratentorial recurrence
KPS: Karnofsky performance status
LMD: Leptomeningeal dissemination
MGMT: O6-methylguanine-DNA-methyltransferase
OS: Overall survival
PFS: Progression-free survival
TERT: Telomerase reverse transcriptase
TMZ: Temozolomide
WHO: World Health Organization
